# Time-resolved transcriptomics reveals mechanisms of acute and sustained low-pH adaptation in *Corynebacterium glutamicum*

**DOI:** 10.1016/j.synbio.2026.03.004

**Published:** 2026-03-23

**Authors:** Yuqing Hao, Jianyu Xu, Bo Sun, Hongyu Zhang, Huifeng Gu, Yongjian Wang, Ping Zheng, Kaizhi Jia, Yan Zhu, Jibin Sun

**Affiliations:** a School of Life and Health Sciences, Hubei University of Technology, Wuhan, 430068, China; b Tianjin Institute of Industrial Biotechnology, Chinese Academy of Sciences, Tianjin, 300308, China; c College of Life Sciences, Nankai University, Tianjin, 300071, China; d University of Chinese Academy of Sciences, Beijing, 100049, China; e School of Biological Engineering, Tianjin University of Science and Technology, Tianjin, 300457, China; f State Key Laboratory of Engineering Biology for Low-Carbon Manufacturing, Tianjin, 300308, China; g National Center of Technology Innovation for Synthetic Biology, Tianjin, 300308, China

**Keywords:** *Corynebacterium glutamicum*, Low-pH stress, Time-resolved transcriptomics, Proton motive force, Cytoplasmic pH homeostasis, β-ketoadipate catabolism, Envelope remodelling

## Abstract

*Corynebacterium glutamicum* is a major industrial cell factory for amino acid production. Acidic byproduct accumulation can lower broth pH, disrupt cytoplasmic pH homeostasis, induce oxidative stress, and ultimately compromise productivity. As low-pH stress is dynamic and process-dependent, distinguishing responses to acute acid shock versus sustained acidification is important for improving strain robustness. Here, we investigated low-pH adaptation under two regimes: acute, unbuffered pH 4.0 shock and sustained pH 5.5 stress with pH control, by integrating viability assays, time-series RNA-seq, and genetic validation. Acute shock caused 93% viability loss within 2 h, followed by broth neutralisation and regrowth, whereas sustained stress led to a 4.1-log_10_ decline over 18 h. Transcriptomics identified 905/847 differentially expressed genes at 1/4 h under acute shock and 643/608/1857 genes at 1/8/18 h under sustained stress. Shared responses included repression of central metabolism and induction of β-ketoadipate catabolism, potassium uptake, sodium/proton antiport, urease-mediated ammonium release, amino acid biosynthesis, and oxidative and membrane-stress defences. Acute shock showed rapid global reprogramming dominated by oxidative protection, chaperone induction, and ion-flux control, whereas sustained stress induced progressive metabolic rewiring, cell envelope reinforcement, and redox buffering. Functional validation using both overexpression and knockout mutants confirmed the contribution of ion transport, iron regulation, β-ketoadipate metabolism, urease, and respiratory modules to low-pH tolerance in a regime-dependent manner. This study provides the first time-resolved, regime-specific transcriptomic dissection of low-pH adaptation in *C. glutamicum* and identifies key modules for engineering acid-resilient strains.

## Graphical abstract

**Figure:**
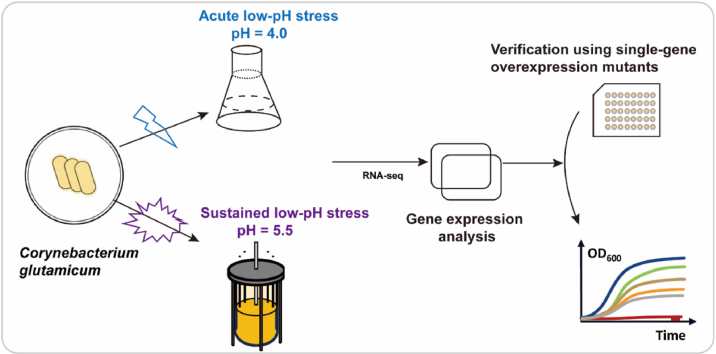

## 1 Introduction

Biomanufacturing underpins sustainable development by enabling low-carbon, high-value production of chemicals, pharmaceuticals, biofuels, and biomaterials [1,2]. *Corynebacterium glutamicum* is a Gram-positive actinobacterium with a mycolic acid-containing cell envelope and serves as a premier microbial cell factory for large-scale production of l-glutamate and l-lysine (together exceeding several million tonnes annually) [3], as well as a growing portfolio of organic acids, alcohols, terpenoids, diamines, and recombinant proteins [4]. Advances in genome editing, systems biology, and synthetic biology have further consolidated its role as a leading microbial chassis [3,4]. During high-density fermentation without effective pH control, accumulation of acidic metabolites (e.g., amino acids and organic acids) can rapidly decrease broth pH (pH_e_), thereby perturbing cytoplasmic pH (pH_i_) homeostasis and compromising productivity [5].

Bacteria employ multifaceted strategies to withstand low-pH stress. These include active removal of excess intracellular protons through F_1_F_0_-ATPase-driven proton efflux, organic acid export, ammonium production, and proton-consuming decarboxylation reactions. In parallel, cells mitigate low-pH induced damage by maintaining the proton motive force (PMF), scavenging reactive oxygen species (ROS), and remodelling the cell envelope [[6], [7], [8], [9]]. Pioneering transcriptomic studies have provided foundational insights into the molecular responses of *C. glutamicum* to pH stress. Long-term adaptation to lactic acid stress revealed significant differential expression of genes related to acid tolerance, transport, and metabolism [10]. A subsequent multi-omics study further indicate that low-pH is coupled to oxidative stress and functional iron limitation, with concomitant TCA cycle perturbation, and induction of methionine/cysteine biosynthesis [11]. *C. glutamicum* grows optimally at pH 7.0–8.5; however, growth declines sharply​ below pH 6.0 and is completely inhibited​ at pH 4.0 [10]. Early microarray and proteomic studies revealed upregulation of metabolic enzymes, cation transporters, and stress-responsive proteins, along with downregulation of F_1_F_0_-ATP synthase at pH 5.7 [12]. A comprehensive multi-omics comparison of pH 6.0 *versus* pH 7.0 identified induction of oxidative stress and iron-starvation responses, activation of methionine and cysteine biosynthesis *via* derepression of the McbR regulon, and suppression of the tricarboxylic acid (TCA) cycle [13,14]. Proteomic analyses further revealed that succinate dehydrogenase and nitrate reductase responded to pH decline [15]. More recently, a single-cell microfluidic study exposed *C. glutamicum* to short pH 4.0–5.0 pulses and observed a surge in intracellular ROS and collapse of pH_i_, followed by heterogeneous recovery across the population. These multi-omics analyses have primarily focused on single timepoints or steady-state comparisons under fixed pH conditions. Consequently, the temporal progression of adaptation and its regulatory coordination remain incompletely resolved. Furthermore, pH-stat cultivation stabilises pH_e_ but continuous titration alters ionic composition, potentially confounding the immediate defences required to counter abrupt proton influxes during uncontrolled fermentation.

To address these limitations, a comparative, time-resolved RNA-seq analysis was conducted to dissect the dynamic responses of *C. glutamicum* under acute pH 4.0 shock without pH control and sustained pH 5.5 stress with pH control. This design enabled direct comparison of immediate shock responses with progressive adaptation during extended acidic exposure. In the early phase, both regimes showed repression of central carbon metabolism and respiration, engagement of the pentose phosphate pathway (PPP), transient induction of β-ketoadipate catabolism, activation of monocation-proton transporters, ammonium buffering, and ROS defences. Divergent responses were observed in the expression of pyruvate kinase, nitrate respiration, and K^+^ transport systems. During the late phase of sustained stress, *C. glutamicum* transitioned to a gene expression programme marked by activation of glycolysis, PPP, and TCA cycle, induction of cytochrome *bd* oxidase and F_1_F_0_-ATP synthase, drastic increases in urease, NAD biosynthesis, sulphur assimilation, and amino acid biosynthesis [arginine, aromatic, and branched-chain amino acids (BCAAs)], repression of β-ketoadipate degradation, enhanced expression of monocation-proton antiporters (*mrp*/*nhaP* Na^+^/H^+^ antiporters, and *kup* K^+^/H^+^ antiporter), and envelope remodelling. Functional validation through mutants confirmed roles of key genes in low-pH tolerance, including *nhaP* and *mrp1* (Na^+^/H^+^ antiporters), *ure* (urease), *pca* (β-ketoadipate metabolism), *lysA* (meso-diaminopimelate decarboxylase), *nar* (nitrate respiration), and *mscL* (mechanosensitive channel). This study provides the first time-resolved comparison of acute and sustained low-pH adaptation in *C. glutamicum*, revealing distinct transcriptional strategies underlying immediate survival and long-term resilience and offering new insights for engineering acid-tolerant industrial strains.

## 2 Materials and methods

### 2.1 Strains and media

*Corynebacterium glutamicum* ATCC 13032 was revived from −80 °C glycerol stock and streaked onto TSBG agar (Tryptone Soya Broth supplemented with 0.5% w/v glucose, pH 7.0). Single colonies were cultured in TSBG at 30 °C, 220 rpm, and subsequently transferred into CGXII minimal medium containing 4% (w/v) glucose. For stress experiments, mid-exponential seed cultures were used to inoculate fresh CGXII medium (details below).

### 2.2 Low-pH conditions

To assess responses to low-pH shock, CGXII medium was pre-adjusted to initial pH 3.0–7.0 in 0.5-unit increments using hydrochloric acid (HCl). Cultures were inoculated at approximately 1 × 10^8^ CFU (colony-forming units) per mL (OD_600_ ≈ 1.0) and incubated at 30 °C, 220 rpm. Broth pH was monitored using a benchtop pH meter (SevenCompact, Mettler Toledo, Shanghai, China). For transcriptomics study, two defined low-pH regimes were applied. For acute shock (non-pH-stat), exponentially growing cells (approximately 1 × 10^8^ CFU/mL) at pH 7.0 were rapidly transferred into pH 4.0 medium, with parallel transfer into pH 7.0 serving as control. For sustained stress (pH-stat), cultures were grown in 5 L bioreactors (Eppendorf, Hamburg, Germany) containing CGXII with 0.1% (v/v) antifoam. A 3-L working volume was inoculated with seed cultures (50 mL TSBG, 500 mL baffled flasks, 30 °C, 220 rpm, 8 h). The bioreactors were maintained at 30 °C, with pH controlled at 5.5 (stress) or 7.0 (control) by automatic acid/base addition. Dissolved oxygen (DO) was regulated by cascade control of agitation and aeration rates.

### 2.3 Viability assays

Cell viability under low-pH stress was assessed by serial dilution and plating, as described previously [16]. For acute shock, samples were collected at 0, 1, 4, and 24 h after transfer to pH 4.0 and pH 7.0. For sustained stress, samples were collected at 0, 1, 8, and 18 h after inoculation into pH-controlled bioreactors (pH 5.5 vs pH 7.0). Samples were serially diluted 1:10 in 0.9% (w/v) saline, and 50 μL aliquots were plated on TSBG agar using an easySpiral 40 automatic spiral plater (Interscience, Saint-Nom-la-Bretèche, France). Plates were incubated at 30 °C for 24 h and viable counts were reported as log_10_ (CFU/mL).

### 2.4 RNA-seq and computational analysis

For acute shock, samples were collected from pH 7.0 (control) and pH 4.0 (stressed) cultures at 1 and 4 h; the 24 h timepoint was excluded because cell regrowth and medium neutralisation confounded the direct transcriptional response to acute low-pH shock. For sustained stress, samples were collected from pH 7.0 (control) and pH 5.5 (stressed) cultures at 1, 8, and 18 h. Cells were pelleted (12,000×*g*, 2 min, 4 °C), supernatants were removed, and pellets were snap-frozen in liquid nitrogen. Total RNA was extracted using a magnetic-bead-based Total RNA Kit (DP761, Tiangen Biotech, Beijing, China); RNA integrity (RIN >8) was verified using a Bioanalyzer 2100 (Agilent Technologies). Libraries were prepared and sequenced on Illumina NovaSeq 6000 platform (paired-end 250 bp; 11.4–17.3 million read pairs per sample; Genewiz, Beijing, China).

Reads were quality-filtered and trimmed using Trimmomatic, assessed with FastQC, and aligned to the *C. glutamicum* ATCC 13032 genome (GenBank GCA_000011325.1) using HISAT2 v2.2.1 [17]. Read summarisation was performed using featureCounts [18]. Differential expression was computed with limma-voom method in Degust, comparing each stress condition against its pH 7.0 control at the same timepoint [19]. Genes with |log_2_ fold change| > 1 and false discovery rate (FDR) < 0.05 were considered differentially expressed. Functional enrichment [Gene Ontology (GO), Kyoto Encyclopedia of Genes and Genomes (KEGG)] and principal component analysis (PCA) were conducted in R.

### 2.5 Reporter feature analysis

Differential expression statistics were integrated with the transcriptional regulatory network from CoryneRegNet 7.0 to identify critical regulatory drivers under low-pH stress [20]. Reporter transcription factors (TFs) were computed following the reporter framework, where the *Z*-score for each TF was defined as *Z* = (*μ*_target_ – *μ*_bg_)/*σ*_bg_, using –log_10_(adjusted *p*) as the measure of gene significance [21]. Regulatory interactions were annotated as activation, repression, or dual regulation, with redundant or ambiguous edges removed. Low-abundance genes (mean counts per million <1) were excluded.

### 2.6 Validation using single-gene overexpression and knockout mutants

Single-gene overexpression strains were selected from an arrayed IPTG (isopropyl-β-D-thiogalactoside)-inducible library and cultivated in CGXII medium with kanamycin (25 μg/mL) (Supplementary Table S1). Where indicated, gene overexpression was induced with 0.05 mM IPTG as described previously [22]. In addition, knockout mutants were constructed *via* suicide plasmid-mediated homologous recombination using pK18mob*sacB*. Briefly, ∼1 kb upstream and downstream homologous arms of each target gene were amplified and ligated into *Bam*HI-linearised pK18mob*sacB* to generate the corresponding knockout plasmids, which were introduced into the parental strain by electroporation to enable allelic exchange. Mutant candidates were verified by colony PCR and further confirmed by Sanger sequencing [23]. Growth validation for both overexpression and knockout strains was conducted under acute and sustained low-pH stress conditions. Growth was monitored in real time using a MicroScreen HT automatic microbial growth curve analyser (Gering Instrument Ltd., Tianjin, China). For acute shock, exponentially growing cells at pH 7.0 (OD_600_ ≈ 1) were rapidly transferred into CGXII at pH 4.0, with parallel transfers into pH 7.0 as controls; for sustained low-pH stress, exponentially growing cells at pH 7.0 (OD_600_ ≈ 1) were rapidly transferred into CGXII adjusted to pH 5.5 [buffered with 39.04 g/L 2-(*N*-morpholino)ethanesulfonic acid, MES; effective buffering range, pH 5.5–6.7; pKa ≈6.1)] and pH 7.0. All cultures were incubated at 220 rpm, 30 °C for 24 h. All experiments were conducted in biological triplicate. The maximum specific growth rate (*μ*_max_) was calculated from the exponential phase as the slope of ln(OD_600_) versus time. Briefly, OD_600_ values were ln-transformed and a sliding 7-point linear regression was performed across the time series. The exponential phase was defined as the contiguous window where R^2^ first reached ≥0.90 and ended when R^2^ dropped below 0.90. *μ*_max_ was taken as the maximum slope among eligible windows. Calculations were performed in Excel.

### 2.7 Fluorescence microscopy imaging

Bacterial cells under low-pH stress were visualised by fluorescence microscopy using propidium iodide (PI) staining, following established protocols [24]. Samples were collected at 2 and 4 h after acute shock (pH 4.0) and initiation of sustained stress (pH 5.5) with control (pH 7.0), pelleted by centrifugation (5600×*g*, 3 min, 4 °C), washed three times with saline (0.1%, w/w), and resuspended to approximately 1 × 10^8^ cells/mL. PI was added to a final concentration of 1 μM, and samples were incubated for 3 min at room temperature in the dark. After staining, cells were washed for three times with saline and then loaded onto glass slides. Images were acquired using a fluorescence microscope (Apotome-3, ZEISS, Germany) equipped with a 63 × oil objective. PI fluorescence was detected at an excitation wavelength of 535 nm and an emission wavelength of 570–620 nm. Bright-field and fluorescence images were captured for the same field of view. Image processing and quantification were performed using ImageJ.

## 3 Results

### 3.1 Growth inhibition, viability loss, and broth alkalinisation after acute low-pH shock

Acute low-pH shock significantly inhibited growth and survival (Fig. 1). As initial pH decreased from pH 7.0 to 3.0, bacterial proliferation progressively declined, with complete growth arrest at pH 3.0–3.5 (Fig. 1A). Kinetic analysis showed that the specific growth rate (*μ*) decreased sharply as pH declined; at pH 4.0, *μ* remained >0.2 h^−1^ up to 8 h before growth ceased, whereas at pH ≤ 3.5 it was near zero (Fig. 1B). OD_max_ peaked at pH 7.0 (31.5), dropped to ∼18 at pH 4.0, and approached baseline at pH ≤ 3.5, while *μ*_max_ was the highest at pH 6.5 (0.44 h^−1^), slightly lower at pH 7.0 (0.42 h^−1^), ∼0.25 h^−1^ at pH 4.0, and close to zero at pH ≤ 3.5 (Fig. 1C). Medium pH showed a biphasic drift (Fig. 1D and E). At initial pH 4.0–7.0, the culture alkalinised to pH 7.0–7.2 within 8 h before re-acidifying to ∼5.5 by 24 h, indicating that *C. glutamicum* actively modified its environment following low-pH shock (Fig. 1D). The largest drift (ΔpH ≈ 3.0) occurred at pH 4.0, likely reflecting active proton-consuming or proton-neutralising metabolic processes during initial adaptation. At initial pH ≤ 3.5, both pH_max_ and ΔpH_max_ were markedly reduced, indicating metabolic collapse (Fig. 1E). Viability assays confirmed these trends (Fig. 1F). At pH 7.0, viable counts increased from 4.1 × 10^8^ to 1.6 × 10^10^ CFU/mL by 24 h, whereas at pH 4.0, they declined by 1.1 log_10_ (∼93% loss) within 2 h and partially recovered to 6.3 × 10^9^ CFU/mL by 24 h (Fig. 1F). The early increase in OD_600_ at pH 4.0 despite reduced CFU suggested accumulation of viable-but-non-culturable (VBNC) cells, dead cells, and cell debris rather than true proliferation. Consistent with this hypothesis, PI staining revealed 62% and 55% PI-positive cells at 2 and 4 h, respectively, following pH 4.0 challenge, whereas virtually none were observed at pH 7.0 (Fig. S1). Acute pH 4.0 shock imposed with either hydrochloric acid (HCl) or sulfuric acid (H_2_SO_4_) produced nearly indistinguishable growth inhibition (Fig. S2). Based on the titration volumes, the associated counter-ion loads were modest, i.e., approximately 6.7 mM Cl^−^ (140 μL of 12 M HCl per 250 mL medium) versus 2.4 mM SO_4_^2−^ (600 μL of 1 M H_2_SO_4_ per 250 mL medium), supporting the conclusion that the phenotype is primarily driven by proton stress rather than anion-specific effects. Collectively, these data define pH 4.0 as a critical threshold for low-pH shock and reveal broth alkalinisation as a hallmark of the early adaptive response.

**Fig. 1:**
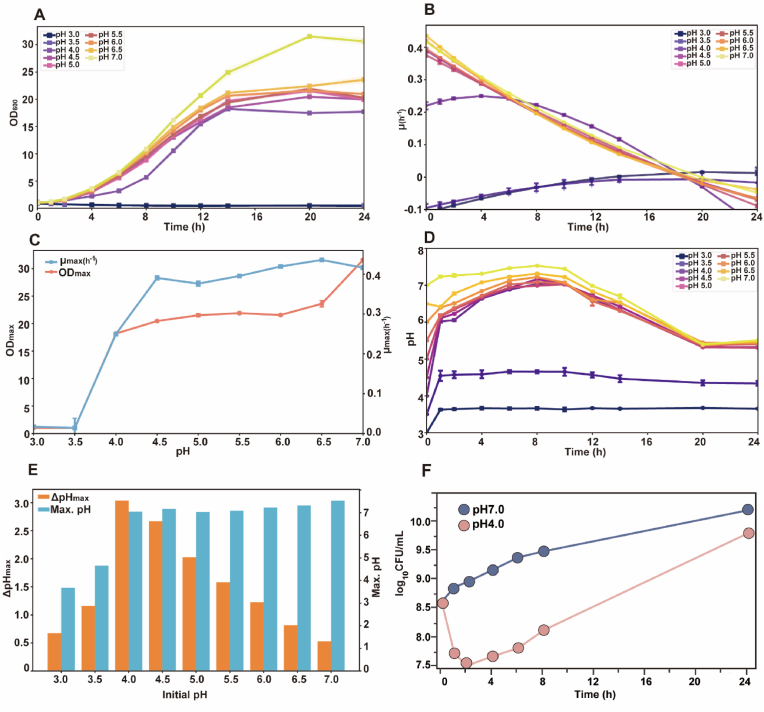
**Growth and mediumpH dynamics of *C. glutamicum* under varying initialpH conditions.** (**A**) OD_600_ profiles monitored over 24 h at varying starting pHs. (**B**) Specific growth rates (*μ*, h^−1^) calculated from OD_600_ data based on spline fitting. (**C**) The maximum OD_600_ and μ at varying starting pH values. (**D**) Temporal variation of medium pH. (**E**) Maximum medium pH and the maximum shifts (ΔpH_max_) at varying starting pHs. (**F**) Viability (CFU/mL) comparison between pH 7.0 and pH 4.0 cultures. Data are shown as mean ± s.d. (standard deviation, *n* = 3) for panels **A**–**D** and **F**.

### 3.2 Constrained growth and metabolism under sustained low-pH stress

Bacterial response to sustained low-pH stress was assessed by comparing parallel batch fermentations at controlled pH 5.5 and 7.0, with dissolved oxygen maintained at ∼30% in both (Fig. 2A). The pH 7.0 fermentation showed transient pH excursions likely due to overtitration, with brief deviations to 3.7 at 3.4 h and 10.7 at 6.6 h, whereas pH 5.5 remained more stable (Fig. 2A). Growth was markedly repressed at pH 5.5 (Fig. 2B and C); final OD_600_ reached 15.9 at pH 5.5 *versus* 53.8 at pH 7.0 (∼70% reduction), and *μ*_max_ decreased from 0.68 h^−1^ (pH 7.0) to 0.34 h^−1^ (pH 5.5, ∼50% reduction) (Fig. 2C). A ∼6 h lag phase preceded slow regrowth at pH 5.5, suggesting metabolic reprogramming before recovery. Viable counts increased from 4.1 × 10^8^ to 2.0 × 10^13^ CFU/mL at 24 h (approximately 4.7-log_10_ increase) for the pH 7.0 control, whereas at pH 5.5 the counts initially declined by 36.5%, then recovered and plateaued near 10^9^ CFU/mL (Fig. 2D). The decoupling of early OD_600_ increase and CFU decline at pH 5.5 again indicates that the optical density captured VBNC cells, dead cells, and debris rather than active cell division and proliferation, which is further supported by PI staining showing 59% and 51% membrane-compromised cells under sustained acidic conditions (Fig. S1). Collectively, sustained pH 5.5 imposed chronic metabolic repression that halved *μ*_max_, reduced final OD_600_ by 70%, and caused a significant adaptation lag, distinct from the acute regime, which caused rapid viability collapse followed by partial recovery *via* environmental neutralisation.

**Fig. 2:**
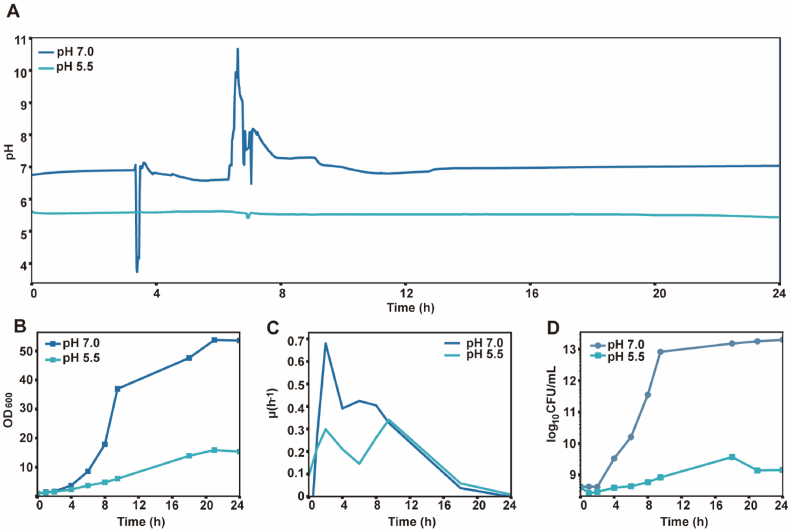
**Growth and mediumpH dynamics of *C. glutamicum* under sustained low-pH stress.** (**A**) Medium pH variation during cultivation at setpoints of 5.5 and 7.0, showing transient deviations from the control value. (**B**) Growth kinetics (OD_600_). (**C**) Specific growth rates (*μ*, h^−1^) calculated from OD_600_. (**D**) Viable cell counts (log_10_ CFU/mL). Data are shown as mean ± s.d. (standard deviation, *n* = 3) for panels **B** and **D**.

### 3.3 Global transcriptional reprogramming

Low-pH exposure induced pronounced, time-dependent transcriptional reprogramming across both regimes. PCA clearly separated stressed samples from pH 7.0 controls, with high replicate consistency (Fig. 3A and B). The number of differentially expressed genes (DEGs) increased over time in both regimes (Supplementary Table S2). Acute shock elicited a rapid and extensive transcriptional response, with 905 DEGs (491 up, 414 down) at 1 h, and 847 (458 up, 389 down) at 4 h relative to pH 7.0 (Fig. 3C). The core set comprised 235 persistently upregulated and 154 persistently downregulated genes, together with 14 that shifted from downregulated at 1 h to upregulated at 4 h and 15 that showed the opposite trend. Sustained stress produced a milder early response that intensified over time, with 643 DEGs (400 upregulated, 243 downregulated) at 1 h, 608 (277 upregulated, 331 downregulated) at 8 h, and 1857 (851 upregulated, 1006 downregulated) at 18 h, and a smaller core set (29 persistently upregulated, 22 persistently downregulated) (Fig. 3D), reflecting dynamic transcriptional reprogramming during adaptation (Supplementary Table S2). At the shared 1 h time point, only 147 genes were co-induced and 105 were co-repressed at pH 4.0 and pH 5.5, defining a narrow core set in the early response to acid stresses, including activation of β-ketoadipate catabolism, cross-membrane transport, and NAD biosynthesis, along with concurrent repression of the TCA cycle, aerobic respiration, Na^+^/H^+^ antiport, and arginine biosynthesis. In contrast, at 1 h, 24 genes were repressed under acute shock but induced under sustained stress, including *eno*, *ppc*, *tpi*, *fda*, *pyk*, and *pgk* from glycolysis, and 27 genes showed the opposite pattern, including *gltBD*, *groESL*, *dnaK*, and Na^+^/solute symporters, indicating regime-specific early allocation of carbon catabolism and ion-transport functions.

**Fig. 3:**
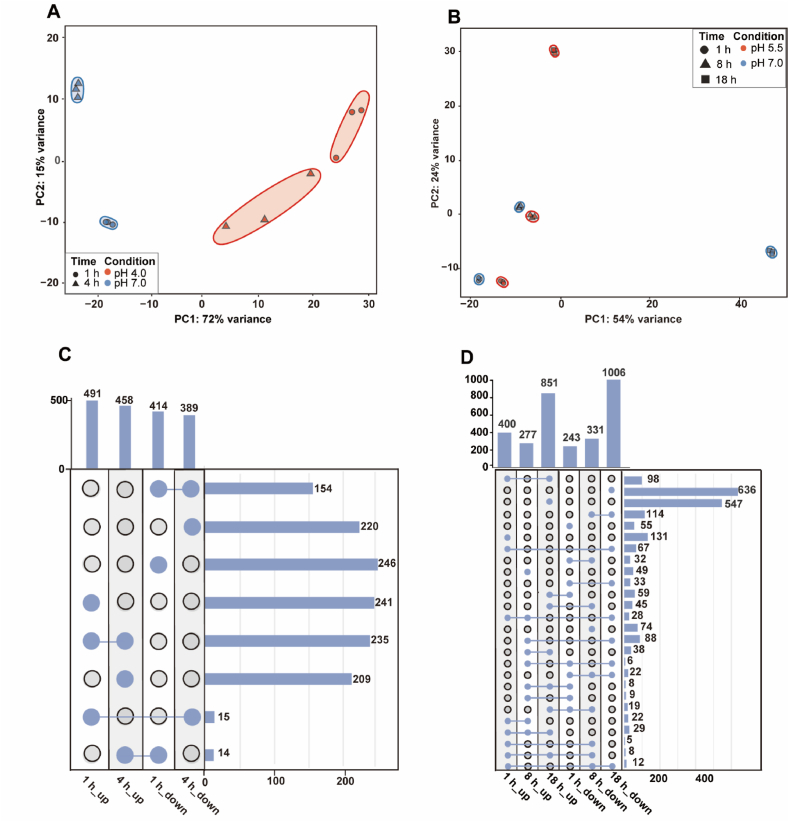
**Global transcriptomic responses to acute and sustained low-pH stresses.** PCA of RNA-seq profiles (**A**, **B**) and UpSet plots of differentially expressed genes (**C**, **D**) relative to pH 7.0; (**A**, **C**) pH 4.0 vs pH 7.0; (**B**, **D**) pH 5.5 vs pH 7.0.

Pathway analysis revealed that acute shock rapidly (1–4 h) increased transcripts associated with aromatic compound degradation, membrane organisation, and transport, while suppressing central carbon metabolism, amino acid biosynthesis, and oxidative phosphorylation, consistent with growth arrest and resource redirection towards stress defence. Under sustained stress, early changes (1 h) included enrichment of benzoate degradation and glycolysis, together with reductions in amino acid and secondary metabolite biosynthesis, carbon and sulphur metabolism, and oxidative phosphorylation (Supplementary Table S3). At 8 h, downregulation centred on ABC (ATP-binding cassette) transporters and propanoate metabolism. By 18 h, translational and cell-envelope functions partially recovered, while metabolism and export processes remained active, indicating progressive recovery under sustained stress. GO term analysis aligned with these trends. Acute stress upregulated stress-response and protein-refolding categories while downregulating glycolysis and amino acid biosynthesis (Supplementary Table S4). Sustained stress showed early enrichment of l-arginine biosynthesis, iron homeostasis, and import pathways, followed by late recovery of translation and cell wall functions. Together, the results indicate distinct temporal responses, characterised by a rapid, defence-centred shutdown upon acute low-pH shock and staged, resource-conserving adaptation under sustained low-pH stress, with membrane, transporter, iron-handling, and ribosomal machinery emerging as core components of the adaptive network.

### 3.4 Transcriptional remodelling of central carbon metabolism

**Fig. 4:**
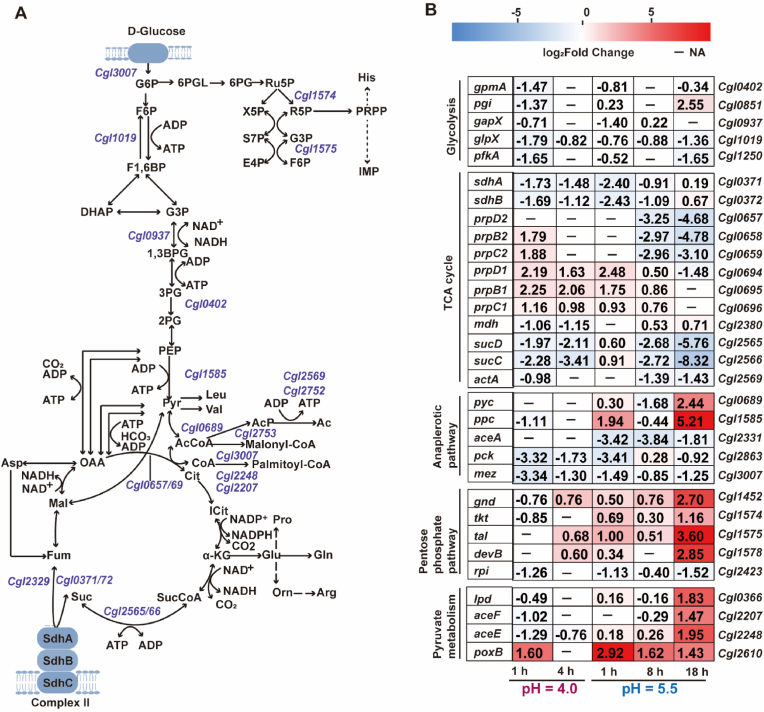
**Central carbon metabolism adaptation of *C. glutamicum* under acute and sustained low-pH stress.** (**A**) Schematic representation of central carbon metabolism, including glycolysis (EMP), tricarboxylic acid (TCA) cycle, pentose phosphate pathway (PPP), anaplerotic reactions (ANAP), and pyruvate dehydrogenase (PDH). (**B**) Heatmaps showing log_2_ fold change in representative genes under during acute (pH 4.0; 1 and 4 h) and sustained (pH 5.5; 1, 8, and 18 h) low-pH stress. 1,3-BPG, 1,3-bisphosphoglycerate; 2PG, 2-phosphoglycerate; 6PG, 6-phosphogluconate; Ac, acetate; AcCoA, acetyl-Coenzyme A; AcP, acetyl phosphate; Arg, arginine; Asp, aspartate; Cit, citrate; E4P, erythrose 4-phosphate; F1,6BP, fructose 1,6-bisphosphate; F6P, fructose-6-phosphate; Fum, fumarate; G6P, glucose 6-phosphate; G3P, glyceraldehyde 3-phosphate; Gln, glutamine; Glu, glutamate; His, histidine; HCO_3_, bicarbonate; IMP, inosine monophosphate; Leu, leucine; Mal, malate; OAA, oxaloacetate; Orn, ornithine; PEP, phosphoenolpyruvate; PRPP, phosphoribosyl pyrophosphate; Pro, proline; Pyr, pyruvate; Ru5P, ribulose 5-phosphate; R5P, ribose 5-phosphate; S7P, sedoheptulose 7-phosphate; Suc, succinate; SucCoA, succinyl-Coenzyme A; Val, valine; X5P, xylulose 5-phosphate.

Acute pH 4.0 shock caused a rapid and broad repression of glycolysis, TCA cycle, anaplerosis, and PPP within 1 h, while selectively inducing the methylcitrate operons (*Cgl0694–96* and *Cgl0657–59*; up to 4.8-fold) (Fig. 4A and B). At 4 h, glycolytic and TCA genes (*pgk*, *gapX*, *sdhCAB*, *sucCD*, *glt*, *fum*) remained downregulated, whereas PPP genes (*zwf*, *gnd*, *devB*, *tal*) showed moderate induction (1.5–2.5-fold), suggesting partial redirection of carbon flux towards NADPH generation for redox buffering.

Under sustained pH 5.5 stress, gene expression followed a phased, adaptive trajectory (Fig. 4B). At 1 h, lower glycolysis was mildly activated (*eno*, *pgk*, *tpi*, *gap*, *pyk*, *glk*, *fda*, 1.3–2.7-fold), while upper glycolytic genes were repressed (*pfkA*, 1.4-fold; *glpX*, 2.6-fold). Phosphoglycerate mutase paralogues diverged, most were downregulated (1.7–3.9-fold), except *Cgl1058* (upregulated 3.7-fold). The TCA cycle was largely repressed (*sdhCAB*, *acn*, *odhA*, *gltA*, *mdh*, *fum*, *icd*, 1.2–5.4-fold), while the methylcitrate operon *Cgl0694–96* was induced by 1.9–5.6-fold and *sucCD* was increased by 1.5–1.9-fold. Anaplerosis was perturbed with *ppc* upregulated by 3.8-fold and glyoxylate shunt downregulated (*aceA* and *aceB*, 1.3–10.7-fold). At 8 h, phosphoglycerate mutase genes were generally downregulated, except *Cgl0438*, which switched from repression to induction (3.3-fold). The oxidative TCA reactions were slightly elevated (*icd* 1.7-fold; *mdh* 1.4-fold), whereas *sdhCAB*, *sucCD*, and the glyoxylate shunt remained repressed (1.9–14.3-fold). By 18 h, broad reactivation of glycolysis (*pgi*, *tpi*, *pgk*, *eno*, *Cgl0438*, *gap*, upregulated by 2.8–8.4-fold) was evident, though *pfkA* and *glpX* remained repressed (3.1- and 2.6-fold). The TCA cycle recovered, with most genes (*gltA*, *acn*, *icd*, *aceF*, *lpd*, *mdh*, *sdhA*, *sdhB*, *odhA*) upregulated by 1.1–5.6-fold; however, *sdhC* remained downregulated (3.0-fold), and succinyl-CoA synthetase persisted as a bottleneck (*sucD* and *sucC* downregulated by 54.3- and 319.9-fold, respectively). Anaplerosis was reconfigured with *ppc* and *pyc* induced (37.2- and 5.4-fold), while *pck* and *aceA* remained downregulated (1.9- and 3.5-fold). The oxidative PPP branch became progressively enhanced under sustained low-pH stress (1.4–2.1-fold at 1 h, 1.2–1.7-fold at 8 h, 2.2–30.5-fold at 18 h), indicating sustained NADPH generation for oxidative defence. Meanwhile, acetate overflow decreased, with *ack* downregulated by 18.8-fold at 18 h and *pta* downregulated by 2.1–23.9-fold at 8–18 h, likely limiting further medium acidification by external acid release (Fig. 4A and B).

### 3.5 Dynamic adjustment of energy metabolism and cross-membrane ion transport

**Fig. 5:**
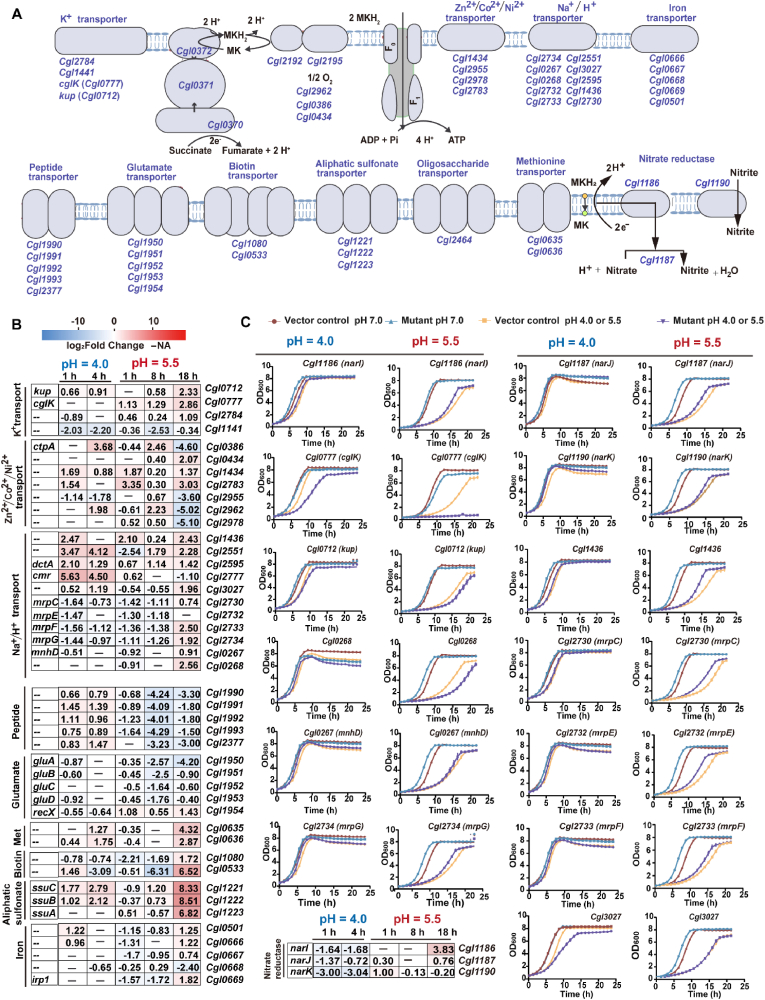
**Energetic and ion-transport adaptation of *Corynebacterium glutamicum* under acute and sustained low-pH stress.** (**A**) Schematic overview of respiratory chain and membrane transporters. (**B**) Heatmap showing log_2_ fold change of representative genes during acute (pH 4.0; 1, 4 h) and sustained (pH 5.5; 1, 8, 18 h) low-pH stress. (**C**) Growth-curve validation of selected metabolic gene overexpression mutants compared with the vector control at acute pH 4.0 shock and sustained pH 5.5 stress. Data are shown as mean ± s.d. (standard deviation, *n* = 3). MK, menaquinone; MKH_2_, menaquinol.

Oxidative phosphorylation is the primary energetic process in aerobic bacteria such as *C. glutamicum*. ATP synthesis is driven by PMF, defined as Δ*ψ* – (2.303*RT*/*F*) ΔpH, where membrane potential Δ*ψ* is negative inside and ΔpH is more alkaline inside. Extracellular acidification increases ΔpH, perturbs PMF, and triggers coordinated transcriptional responses [25]. Aerobic respiratory genes were rapidly repressed under both low-pH regimes. At 1 h after pH 4.0 shock, the expression of complex II (*sdhCAB*), the cytochrome *bc*_1_*-aa*_3_ oxidase supercomplex (complex III/IV, *qcr* and *cta* genes), and F_1_F_0_-ATPase (*atp* operon) decreased by 1.6–4.8-fold, and the repression persisted to 4 h (Fig. 5A). Under sustained pH 5.5 stress, most respiratory genes were downregulated by 1.2–5.4-fold at 1 h, except *cydA* and *cydB* (cytochrome *bd* oxidase). At 8 h, the repression was lessened, with *cyd* genes upregulated by 2.9–3.3-fold. Moreover, nitrate reductase genes (*narHIJK*) were upregulated by 1.2–2.0-fold at 1 h and 1.2–14.2-fold at 18 h. Individual overexpression of *narIJK* increased tolerance to low-pH stress (Fig. 5C–Supplementary Table S5). By 18 h, the *atp* operon was drastically upregulated (1.2–32.7-fold), the *bd* oxidase increased further (1.9–11.6-fold), and components of complexes II and III/IV recovered (1.1–7.7-fold), although *sdhC* and *ctaE* remained repressed by 3.0- and 3.7-fold, respectively. These dynamics demonstrate a regime-specific energy adaptation, with shock-induced respiratory arrest *versus* stress-driven energetic recovery phase that optimises proton translocation and ATP yield under low-pH conditions.

Ion transporters were extensively regulated to stabilise PMF (Fig. 5A and B). The Kef-type K^+^ uniporter *cglK* (*Cgl0777*) was selectively upregulated (2.2–7.2-fold) under sustained stress but remained unchanged after acute shock, while the high-affinity K^+^ importer *kup* (*Cgl0712*) increased modestly during acute shock (1.6–1.9-fold) and was progressively induced (2.5–5.1-fold) under sustained stress. Among putative flavoprotein partners associated with K^+^ transporters, *Cgl2783-85* were upregulated under both low-pH conditions, whereas *Cgl1141*, *Cgl1509*, and *Cgl2887* were repressed. Overexpression of *cglK* or *kup*, however, failed to improve tolerance; *cglK* markedly increased susceptibility, whereas *kup* caused only a slight increase (Fig. 5C–Supplementary Table S5). Although *cglK* deletion elevated acid susceptibility [25], excessive K^+^ uptake provided no benefit. Effective acid adaptation requires a finely balanced K^+^ influx, sufficient to stabilise Δψ without dissipating PMF; surpassing this range likely disrupts Δψ/ΔpH homeostasis and compromises energetic efficiency under low-pH stress.

Sodium-linked transport displayed distinct temporal regulation. Acute pH 4.0 shock rapidly induced several Na^+^-coupled uptake systems, notably *Cgl2551* (*dctA* homolog, 11.1–17.5-fold), *dctA* (*Cgl2595*, 2.5–4.3-fold), *Cgl1005* (4.1-fold at 1 h), *Cgl0206* (1.5–2.8-fold), *Cgl2327* (2.2–3.1-fold), *Cgl1436* (*nhaP*, 5.6-fold at 1 h), and *Cgl3027* (1.4–2.3-fold), while repressing multi-subunit *mrp* Na^+^/H^+^ antiporters (*mrp1*, *Cgl2729-34*; *mrp2*, *Cgl0269-64*; 2.7–3.1-fold). Adjustment with 140 μL of 12 M HCl per 250 mL medium corresponds to approximately 6.7 mM added Cl^−^ and does not introduce Na^+^, supporting the interpretation that induction of Na^+^-linked systems reflects acid stress associated ion homeostasis rather than Na^+^ addition during titration. Under sustained pH 5.5 stress, Na^+^-solute symporters were broadly repressed at 8 and 18 h, including *putP* (4.3–4.8-fold), *Cgl1005* (2.9–12.4-fold), and *Cgl0833* (1.2–8.9-fold), whereas Na^+^/H^+^ antiporters recovered late at 18 h, including *mrp* genes (1.7–5.9-fold) and *Cgl1436* (5.4-fold). *Cgl2551* was transiently suppressed at 1 h (5.8-fold) but re-induced by 8–18 h (3.5–4.9-fold). Overall, cells appeared to prioritise Na^+^-coupled nutrient uptake, repress *mrp* Na^+^/H^+^ antiporters and upregulate *nhaP* immediately after acute shock, whereas under sustained stress cells repressed Na^+^-solute symporters and then restored Na^+^ extrusion through late induction of *nhaP* and *mrp* together with *mrp*-like components as homeostasis was re-established. Individual overexpression of *mrp1* subunits (*mrpEFG*, *Cgl2732-34*) and *mrp2* component *mnhD* (*Cgl0267*) enhanced tolerance under both low-pH regimes, whereas *mrpC* (*Cgl2730*) improved tolerance exclusively to sustained pH 5.5 stress, and *Cgl0268* showed no detectable effect (Fig. 5C–Supplementary Table S5). These results suggest that the *mrp1* operon is the dominant Na^+^/H^+^ antiporter system for acid resistance in *C. glutamicum*, extending previous transcriptional observations [15,25]. Overexpression of *nhaP* also enhanced tolerance to low-pH stress, whereas *Cgl3027* overexpression produced insignificant effect (Fig. 5C–Supplementary Table S5).

Heavy-metal cation transporters (*Cgl1434*, *Cgl2783*, and *Cgl2133*) were induced under both low-pH conditions. Several energy-intensive P-type ATPases including *copA* (*Cgl0386*), *copB* (*Cgl2962*), *ctpC* (*Cgl1176*), *pacL* (*Cgl1546*), and *Cgl2974*, showed varying responses to acute shock and transient induction followed by repression under sustained low-pH stress, likely mediating early metal detoxification before being downregulated to conserve ATP once pH homeostasis was re-established.

### 3.6 Activated β-ketoadipate catabolism

**Fig. 6:**
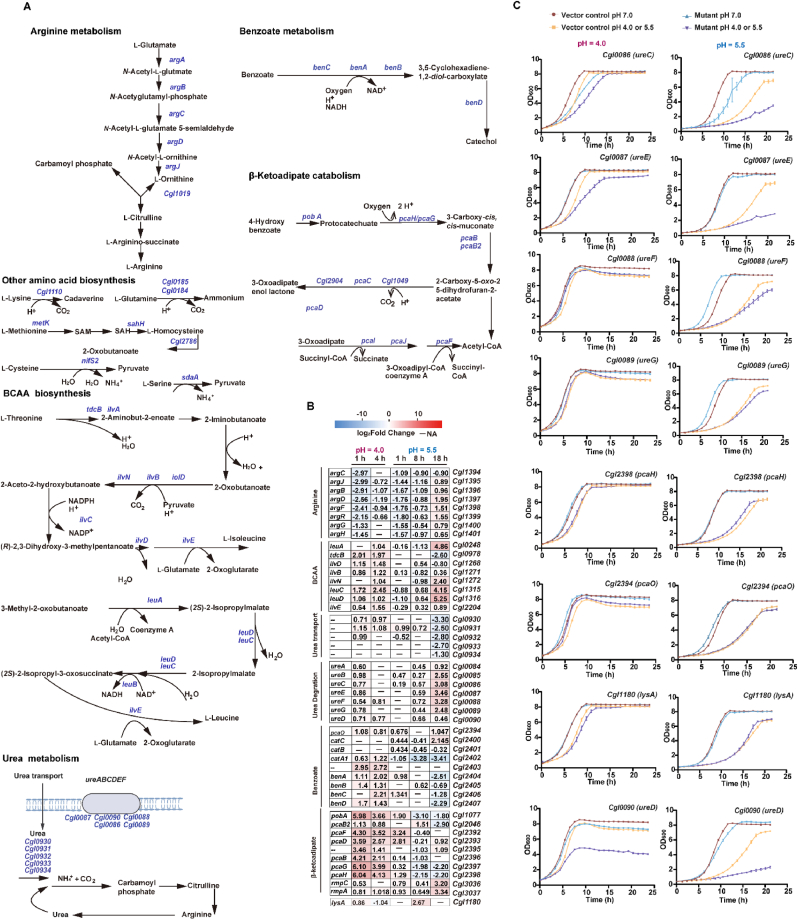
**Amino acid, urea, and aromatic metabolism contributing to low-pH adaptation.** (**A**) Schematic overview of amino acid, urea, and benzoate/β-ketoadipate metabolic pathways. (**B**) Heatmap of representative genes showing log_2_ fold changes during acute (pH 4.0; 1, 4 h) and sustained (pH 5.5; 1, 8, 18 h) stress. (**C**) Growth-curve validation of selected metabolic gene overexpression mutants compared with the vector control at acute pH 4.0 shock and sustained pH 5.5 stress. Data are shown as mean ± s.d. (standard deviation, *n* = 3). BCAA​, branched-chain amino acids; CoA, coenzyme A; SAM, *S*-adenosylmethionine; SAH, *S*-adenosylhomocysteine.

Both regimes activated the β-ketoadipate pathway, but with distinct timing and intensity. Acute shock induced a rapid, high-amplitude upregulation of *pcaIJ* (*Cgl2389–90*), *pcaRFDO* (*Cgl2391–94*), *pcaHGBC* (*Cgl2398–95*), *pobA* (4-hydroxybenzoate 3-monooxygenase, *Cgl1077*), and *Cgl2*904 by 2.1–68.7-fold at 1 h and 1.8–20.7-fold at 4 h (Fig. 6A and B). Under sustained pH 5.5 stress, *pca* genes showed only modest induction at 1 h (1.8–16.2-fold) followed by repression by 8 h (1.1–4.4*-*fold); *pobA* was upregulated by 8.6-fold but declined thereafter despite persistent extracellular acidity. These dynamics suggest a pH-dependent effect, whereby stronger acid shock elicited an immediate and more pronounced upregulation of *pca* and *pobA*. Interestingly, aromatic amino acid biosynthesis genes (*trpEGDCFBA*, *aroCKB*, and *aroE3*) were repressed early under both acute and sustained low-pH stress, but were significantly upregulated at 18 h of sustained low-pH stress (1.9–12.1-fold).

Functional validation highlighted distinct roles of *pcaO* (*Cgl2394*) and *pcaH* (*Cgl2398*) in low-pH adaptation. Overexpression of *pcaO*, which encodes a LuxR-family transcriptional activator reported to govern expression of the *pcaHGBC* operon (*Cgl2398-95*) and *pobA-pcaK* (*Cgl1077-76*), produced only a modest fitness gain against acute low-pH shock (Fig. 6C–Supplementary Table S5), whereas overexpression of *pcaH* encoding the β subunit of protocatechuate 3,4-dioxygenase that catalyses aromatic ring cleavage, conferred a growth benefit specifically during prolonged exposure to pH 5.5 (Fig. 6C–Supplementary Table S5). Together, these data support a division of labour in which PcaO primarily tunes pathway induction, while PcaH determines pathway flux capacity, explaining their distinct phenotypic impacts under acute versus sustained low-pH stress and providing direct experimental evidence that β-ketoadipate catabolism contributes critically to acidic pH tolerance in *C. glutamicum*.

### 3.7 Perturbed amino acid metabolism

Arginine catabolic genes (*argCJBDFR*, *Cgl1394-99*; *argGH*, *Cgl1400-01*) were downregulated early (2.5–8.0-fold and 1.6–2.3-fold at 1 and 4 h after acute shock, respectively; 1.5–3.5-fold at 1 h of sustained stress), but showed late induction at 18 h under sustained stress (1.6–3.9-fold, Fig. 6A and B). The *ureABCEFGD* operon (*Cgl0084-90*) was modestly upregulated at 1 h after acute shock (1.5–2.0-fold) and under sustained stress (1.2–1.7-fold) then progressively increased to 1.4–11.0-fold at 18 h during sustained stress, suggesting enhanced urea degradation and ammonium release for pH_i_ neutralisation. Additionally, urea uptake genes (*urtABC*, *Cgl0930–32*) were upregulated (1.6–2.2-fold, 1–4 h) upon acute shock; *urtB* was increased (1.6–2.0-fold) during sustained stress over 1–8 h, whereas the *urt* operon (*Cgl0930–34*) was significantly repressed by 2.5–10.0-fold at 18 h. However, individual overexpression of *Cgl0086-90* failed to improve growth but generally reduced fitness under either sustained pH 5.5 stress or acute pH 4.0 shock, despite showing little effect at pH 7.0 (Fig. 6C–Supplementary Table S5). This pH-dependent cost suggests that increased *ure* expression is beneficial only when it increases net buffering flux, but under low-pH stress it more often adds metabolic/proteostasis burden and perturbs nitrogen/pH homeostasis when energy and PMF are already limiting. Consistent with this, overexpression of the maturation factors *ureF* (*Cgl0088*) and *ureG* (*Cgl0089*) rather than structural components provided the clearest benefit following pH 4.0 shock (Fig. 6C–Supplementary Table S5), implying that improving enzyme activation can be more effective than increasing component abundance. Branched-chain amino acid (BCAA) biosynthesis genes (*tdcB*, *liv*, *leu*) were broadly induced by 1.6–5.5-fold after acute shock, and surged at 18 h of sustained stress (*leuCD* 17.7–38.0-fold, *leuA* 28.9-fold, *ilvN* 5.3-fold, *tdcB* 5.9-fold). These responses likely increased branched acyl-CoA availability for mycolate biosynthesis and redirected carbon flux away from acidifying overflow metabolism (Fig. 6A and B).

Sulphur amino acid metabolism was modestly activated under acute shock but massively induced under prolonged acidity, with major increases of *metE* (*Cgl1139*, 47.1-fold), *metH* (*Cgl1507*, 14.7-fold), *metXY* (*Cgl0653-52*, 3.7–14.8-fold), *nifS2* (*Cgl1561*, 26.4-fold), *cysJ* (13.7-fold), and *cysIHDN*–*Cgl2813*–*2* (1.6–103.3-fold) (Fig. 6A and B), suggesting increased thiol-based redox buffering to counter oxidative stress accompanying low pH. Additionally, lysine metabolism was activated under low-pH stress. Expression of *Cgl1110* (lysine decarboxylase family protein) increased 1.8-fold (acute 4 h) and 2.5-fold (sustained 1 h) (Fig. 6A and B). In parallel, *lysA* (*Cgl1180*), which encodes diaminopimelate decarboxylase catalysing the final step of lysine biosynthesis, increased 1.8-fold (acute 4 h) and 6.4-fold (sustained 18 h), suggesting that enhanced lysine supply may support cell envelope integrity during prolonged acid stress (Fig. 6A and B). Consistently, *lysA* overexpression conferred a modest improvement in tolerance to sustained low-pH stress (Fig. 6C–Supplementary Table S5).

### 3.8 Oxidative and envelope responses

**Fig. 7:**
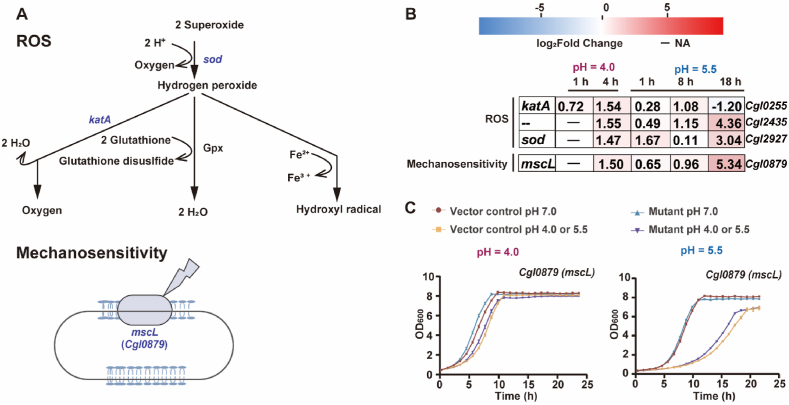
**ROS and mechanosensitive ion channel contributing to low-pH adaptation.**​​ ​​(**A**) Schematic overview of ROS and mechanosensitive ion channel pathways. (**B**) Heatmap of representative genes showing log_2_ fold changes during acute (pH 4.0; 1, 4 h) and sustained (pH 5.5; 1, 8, 18 h) stress. (**C**) Growth-curve validation of selected metabolic gene overexpression mutants compared with the vector control at acute pH 4.0 shock and sustained pH 5.5 stress. Data are shown as mean ± s.d. (standard deviation, *n* = 3). ROS, reactive oxygen species.

Following acute pH 4.0 shock, antioxidant genes were rapidly induced (Fig. 7A and B), including *sod* (superoxide dismutase, *Cgl2927*, 2.8-fold at 4 h), *katA* (catalase, *Cgl0255*, 1.7-fold at 1 h and 2.9-h fold at 4 h), and *ahpD* (peroxidase, *Cgl2435*, 2.9-fold at 4 h). Under sustained pH 5.5 stress, *sod* showed progressive induction (1.1–8.2-fold), *katA* peaked at 8 h (2.1-fold), and then declined at 18 h (2.2-fold), and *ahpD* remained consistently upregulated (1.4–20.6-fold). Acute shock also activated organosulphur metabolism, with strong induction of the alkanesulphonate uptake/oxidation systems (*ssuD1CBA*, *ssuD2*, and *ssiEFG*, 2.5–7.3-fold at 4 h). Under sustained pH 5.5 stress, these genes were transiently repressed at 1 h, followed by striking upregulation at 18 h (*ssu*, 113–419-fold; *ssi*, 24–292-fold), indicating a metabolic shift toward organosulphur utilisation during prolonged acidity. The *Cgl1069-67* operon and *Cgl1070* (ADP-ribose pyrophosphatase) were also markedly induced at 18 h (4.4–26.8-fold), suggesting late reinforcement of NAD biosynthesis and redox buffering capacity.

Acute pH 4.0 shock predominantly repressed mycolate and envelope biosynthesis genes (Fig. 7A and B), whereas sustained pH 5.5 stress caused early repression (1–8 h) followed by marked induction at 18 h, including *pks* (*Cgl2871*, polyketide synthase, 3.9-fold), *fadD* (*Cgl2872*, acyl-AMP ligase, 4.6-fold), and *accD2* (*Cgl0707*, acyl-CoA carboxylase β subunit, 3.1-fold). Corynomycolyl transferases displayed divergent regulation. Acute shock downregulated *cmt1* (*Cgl0343*, 3.6-fold) and *cmt2* (*Cgl2878*, 6.3-fold) while transiently inducing *cmt5* (*Cgl1031*, 2.8-fold at 1 h), whereas sustained low-pH stress caused late upregulation of *cmt1* (2.4-fold), *cmt2* (5.2-fold), and *cop1* (*Cgl2875*, trehalose corynomycolyl transferase, 1.9-fold) at 18 h. Mechanosensitive channels were upregulated under both conditions, including *Cgl1270* (*mscS*) upregulated 6.3–9.2-fold for acute stress, 2.0–8.0-fold for sustained stress, and *Cgl0879* (*mscL*) upregulated 2.8-fold at 1 h of acute stress, and 1.6–40.5-fold for sustained stress. Overexpression of *mscL* enhanced tolerance to sustained low-pH stress (Fig. 7C–Supplementary Table S5). The membrane insertase *yidC* was induced 1.9-fold at 1 h under acute shock and 1.4–1.7-fold during sustained stress. The cyclopropane fatty acid synthetase *Cgl0573* increased 1.9–3.2-fold and 4.6–21.6-fold under acute shock and sustained stress, respectively. *Cgl1439* (membrane hydrolase) rose 2.0-fold (acute 1 h) and 8.4–33.9-fold (sustained 8 and 18 h), implying a key role in envelope maintenance under low-pH stress (Fig. 7A). Acute pH 4.0 triggered a drastic upregulation of chaperones including *dnaK-grpE, dnaJ*, *dnaJ2*, *groES, groEL*, *clpB*, and *clpC* (3.8-39.7-fold), whereas sustained pH 5.5 initially suppressed these genes before a late rebound at 18 h. Proteases were also activated, with the periplasmic trypsin-like protease *Cgl0*876 most prominently upregulated (40.9-fold at 1 h after acute shock).

### 3.9 Significantly perturbed regulators

**Fig. 8:**
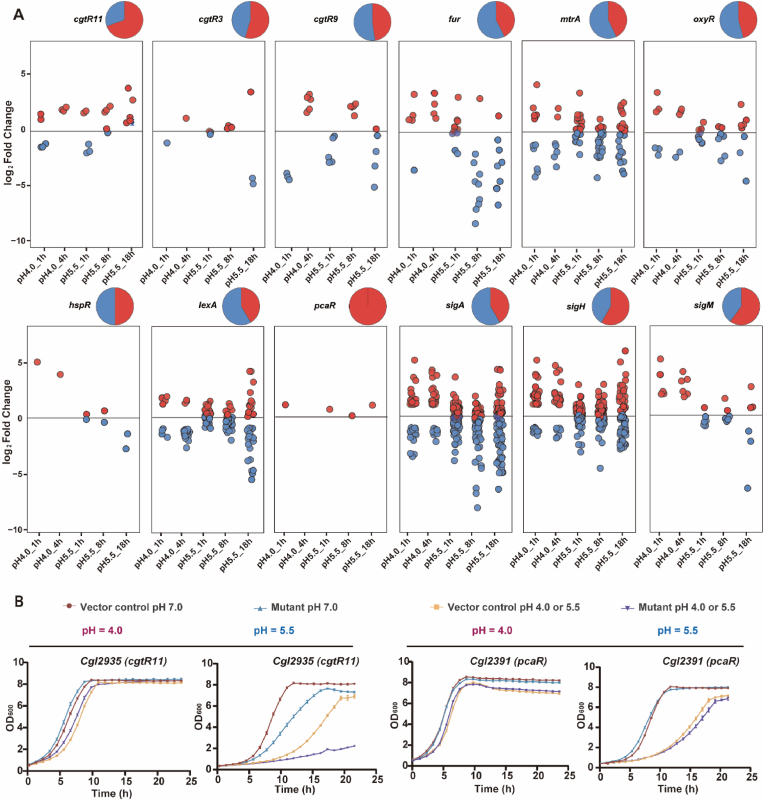
**Transcriptional regulators mediating low-pH adaptation.** (**A**) Time-resolved expression profiles of key transcriptional regulators under acute (pH 4.0) and sustained (pH 5.5) acid stress relative to neutral conditions. Each panel shows log_2_ fold changes of the target genes over time, with red and blue denoting upregulation and downregulation, respectively. Pie charts summarise the proportions of regulated targets for each regulator. (**B**) Growth-curve validation of selected metabolic gene overexpression mutants compared with the vector control at acute pH 4.0 shock and sustained pH 5.5 stress. Data are shown as mean ± s.d. (standard deviation, *n* = 3).

Reporter feature analysis identified 34 transcriptional regulators significantly perturbed during low-pH adaptation (Fig. 8A). Among these, 27 and 24 regulators were affected at 1 and 4 h, respectively, after acute shock, and 29, 26, and 22 were affected at 1, 8, and 18 h, respectively, under sustained stress. A core module comprising *sigH* (oxidative/heat-stress), *sigA* (housekeeping), *oxyR* (activator of antioxidant defence), *hspR* (*Cgl2797*, repressor of heat-shock chaperones), and *cgtR11* (*Cgl2935*, regulator of haem uptake and utilisation) was shared between both regimes. Acute shock transiently activated *hspR* (11.0-fold at 1 h) and *sigH* (2.1-fold at 4 h) with modest upregulation of *sigA*, whereas sustained stress caused a brief *sigH* increase (1.3-fold at 8 h) before repression at 18 h (4.0-fold) accompanied by reduced expression of *hspR* and *sigA*. Under both regimes, *cgtR11* and *oxyR* were persistently downregulated, suggesting attenuated haem/iron uptake signalling and induction of antioxidant genes as shared stress responses.

Acute shock uniquely activated *pcaR* (30.0-fold at 1 h, 11.9-fold at 4 h), *cgtR3* (stress-responsive, 10.4- and 4.1-fold), and *sigM* (envelope biogenesis and stress, 2.6- and 1.9-fold) while repressing *argR*, *fur*, *lexA*, and *whiB4* (redox-responsive Fe–S transcription factor). At 1 h under sustained stress, *Cgl0010* (putative activator of *ssi* and *ssu* genes) and *Cgl0121* (putative regulator of *cysIHDN*–*Cgl2813*–*12*) were repressed by 9.4- and 15.7-fold, respectively. By 18 h, *sigM*, *whiB4*, *cgtR9* (membrane regulator), and *fur* (*Cgl2280*) were significantly downregulated (2.4–64.4-fold), while *Cgl0010*, *mtrA*, *Cgl1070* (putative regulator of *nadAC*–*Cgl1067* operon), *argR*, and *pcaO* were upregulated, underscoring their roles in adaptation to low-pH stress (Fig. 8A). Overexpression of *cgtR11* improved tolerance to acute low-pH shock but reduced fitness under sustained low-pH stress, whereas *pcaR* overexpression provided no significant benefit during acute shock and decreased tolerance during sustained low-pH exposure (Fig. 8B–Supplementary Table S5). Consistent with a repressive role for PcaR, deletion of *pcaR* (*Cgl2391*) increased tolerance under sustained pH 5.5 (Fig. S3, Supplementary Table S5), likely by derepressing the β-ketoadipate module (including *pcaO*) and thereby strengthening aromatic detoxification capacity during prolonged acidity. Deletion of *fur* resulted in growth failure under acute stress, indicating that Fur is required for survival under rapid acidification (Fig. S3, Supplementary Table S5). This likely reflects loss of iron homeostasis under low-pH conditions, which can amplify ROS-mediated damage and prevent recovery. Together, these results reveal temporally distinct transcriptional control of networks governing envelope stability, redox and metal homeostasis, amino acid biosynthesis, and chaperone systems during acid stress.

## 4 Discussion

Industrial fermentation frequently exposes microorganisms to dynamic low-pH stress arising from acidic byproduct accumulation, imbalance in ammonium assimilation, imperfect mixing, and delayed or uneven titration [1]. These processes generate transient pH drops followed by sustained moderate acidity, creating fluctuations that severely challenge intracellular homeostasis [25]. To reflect these two regimes, we selected pH 4.0 as an acute shock condition and pH 5.5 as a sustained, sublethal mild acid stress that permits long-term adaptation (Fig. 1, Fig. 2).

This experimental design complements earlier studies by explicitly separating acute shock from sustained adaptation. Prior work examined acute low-pH responses under unbuffered, lactate-based conditions, whereas long-term pH adaptation was analysed under buffered cultivation across a broad pH range with pH adjusted using H_2_SO_4_ [10,11]. Consistent with the acute-stress study, we observed rapid growth collapse at highly acidic pH, supporting pH 4.0 as a critical shock threshold, while pH 5.5 lies near the lower boundary of sustainable stress and is therefore suitable for modelling long-term adaptation (Fig. 1, Fig. 2) [10,11,24]. Acid identity and medium composition also differ across studies and can shape response patterns. Lactate may introduce weak-acid effects beyond proton stress and was tested in rich TSB medium, whereas buffer-controlled pH adaptation was conducted in a low-carbon minimal medium containing 1% glucose and substantially lower nitrogen availability relative to CGXII [10,11]. Here, we used CGXII with 4% glucose and inorganic acids (HCl, validated with H_2_SO_4_), enabling clearer attribution of responses to external proton concentration under fermentation-relevant growth conditions. Parallel HCl and H_2_SO_4_ treatments produced highly similar growth profiles (Fig. S2), indicating that the observed responses were primarily proton-driven rather than by the conjugate anion, in agreement with reports that proton concentration is the dominant trigger of generalised acid stress in many bacteria [26,27].

As a neutrophilic bacterium, *C. glutamicum* requires tight regulation of cytoplasmic pH (pH_i_ approximately 7.5–7.7), and even modest deviations can disrupt enzyme catalysis, impair PMF, and compromise overall bioenergetic efficiency [11]. Additionally, acidification causes multiple cellular stresses, including membrane destabilisation, iron dysregulation, and oxidative damage, collectively impairing growth and viability (Fig. 1, Fig. 2).

Bacteria employ diverse protective strategies to mitigate low-pH stress [28]. Na^+^/H^+^ antiporters (e.g., *nhaA*, *nhaB* in *E. coli*, and *mrp* operons in *Bacillus subtilis*) expel protons in exchange for extracellular Na^+^, thereby stabilising pH_i_ [[29], [30], [31], [32]]. Amino acid decarboxylases consume protons while generating alkaline products such as γ-aminobutyric acid (glutamate decarboxylase), agmatine (arginine decarboxylase), or cadaverine (lysine decarboxylase) to neutralise cytoplasmic acidity [[33], [34], [35], [36], [37]]. Urease hydrolyses urea to release ammonium and CO_2_, buffering the cytoplasm [38,39]. Proton-translocating efflux pumps export organic acids, while membrane remodelling *via* cyclopropane fatty acid synthase or mycolic acid modification reduces proton permeability [[40], [41], [42], [43], [44], [45]]. Together, these mechanisms preserve PMF and pH_i_ homeostasis under acidic conditions.

Previous studies indicate that *C. glutamicum* maintains pHi largely through ion-driven control of membrane potential, primarily mediated by Na^+^/H^+^ antiporters (*nhaP*, *mrp1*, *mrp2*) and K^+^ transporter (*cglK*, *kup*) [25]. Multi-omics studies further suggest that low-pH responses are tightly coupled to redox and metal homeostasis. Acidic conditions provoke oxidative stress and functional iron limitation, triggering metabolic rewiring and induction of sulphur metabolism (methionine and cysteine biosynthesis), alongside activation of global stress regulation (e.g. SigB/SigE) and potential post-translational control [10,11]. These findings indicate that low-pH adaptation extends well beyond direct proton management (Fig. 3).

Consistent with earlier reports, our data reveal upregulation of *sigB*/*sigE* (Supplementary Table S2), induction of oxidative-defence programmes, perturbation of iron/sulphur-associated metabolism, and engagement of ion transport systems that maintain pH_i_ and PMF. Extending these findings, our time-resolved transcriptomic datasets reveal coordinated induction of K^+^ uptake (*cglK*, *kup*), Na^+^/H^+^ exchange, β-ketoadipate catabolism, urease-mediated ammonium release, lysine-associated buffering reactions, and oxidative/membrane-stress defences across acute and sustained regimes (Fig. 4, Fig. 5, Fig. 6, Fig. 7). Importantly, functional validation using overexpression or knockout mutants confirmed the involvement of 18 out of 26 tested genes in low-pH tolerance, including *nhaP* and *mrp1* (Na^+^/H^+^ antiporters), *ure* (urease), *pca* (β-ketoadipate metabolism), *fur* (iron utilisation), *lysA* (meso-diaminopimelate decarboxylase), *nar* (nitrate respiration), *mscL* (mechanosensitive channel) (Fig. 4, Fig. 5, Fig. 6, Fig. 7 and S2, Supplementary Table S5).

A key finding of this study is the previously unrecognised link between β-ketoadipate catabolism and low-pH tolerance.​ The *pca* genes were significantly upregulated upon acute shock and at the onset of sustained stress (Fig. 5A and B), suggesting early pathway activation during adaptation. One plausible role is redox protection. The β-ketoadipate pathway catabolises redox-active aromatics (e.g., catechol and protocatechuate), which in other contexts may participate in redox cycling and promote ROS formation. This interpretation is supported indirectly by prior observations that H_2_O_2_-tolerant *C. glutamicum* strains constitutively upregulate the *pca* operon [46], that *pca* overexpression can enhance resistance to peroxide stress [47], and that *pca* genes are induced during H_2_O_2_ exposure [16]. However, our targeted metabolomics (data not shown) did not detect clear accumulation of aromatic intermediates under low pH. Therefore, while the genetic and transcriptional data support involvement of the *pca* module in acid tolerance, the proposed role in aromatic detoxification or ROS suppression remains a plausible mechanism that warrants dedicated investigation in future studies. Under acidic conditions, increased iron solubility may nevertheless elevate the potential hydroxyl radical formation through Fenton chemistry, which could increase the selective value of pathways that support redox and metal homeostasis.

Functional genetics support an involvement of the *pca* module in low-pH tolerance. Overexpression of *pcaH* enhanced survival under sustained pH 5.5 stress, whereas overexpression of the transcriptional activator *pcaO* increased tolerance to acute low-pH shock, suggesting that increased flux capacity may be more beneficial during prolonged exposure while rapid pathway activation is advantageous during acute shock (Fig. 6C–Supplementary Table S5). Mechanistically, PcaO activates *pcaHG* operon, and its DNA binding is weakened by ATP but strengthened by ADP, linking induction to the low-energy state typical of acute acid stress [48]. As cellular energy charge recovers during sustained exposure to low pH, *pca* induction would be expected to decline, consistent with the observed late downregulation (Fig. 6A and B). Knockout phenotypes further highlight the importance of coordinated aromatic catabolism and iron regulation (Fig. S3). Deletion of *fur*, a global iron-responsive regulator, caused a complete loss of growth following acute pH 4.0 shock while growth at pH 7.0 remained largely comparable to vector control, consistent with disrupted iron homeostasis exacerbating ROS stress under severe acid stress. In contrast, deletion of *pcaR*, a transcriptional repressor of the β-ketoadipate pathway, increased tolerance under sustained pH 5.5 stress, likely through derepressing *pca* expression during prolonged exposure (Fig. S3). Together, these results implicate coordinated regulation of iron availability (Fur) and β-ketoadipate pathway (PcaR/PcaO) as contributors to low-pH adaptation in *C. glutamicum*.

Previous work links the urease operon (*ureABCEFGD*, *Cgl0084–90*) and urea transport (*urtABCDE*, *Cgl0930–94*) to AmtR-mediated nitrogen control, with induction under nitrogen limitation rather than low-pH stress and, in *C. glutamicum*, with no prior functional evidence for a role in low-pH tolerance. In the present study, both *urt* and *ure* genes were induced at low pH, and overexpression of urease components (*ureF*, *ureG*) increased survival during low-pH stress, consistent with ammonium-mediated pH_i_ buffering (Fig. 6). Although urease-dependent acid resistance is established in other bacteria (e.g., *Helicobacter pylori*, *streptococci*), it has not been previously demonstrated in *C. glutamicum*; thus, these results provide, to our knowledge, the first functional evidence that urea uptake and urease constitute an acid-defence module in this microorganism, complementing cation–proton homeostasis. Furthermore, transcriptomics showed induction of *Cgl1110*, *lysA*, and *fdh* under both low-pH regimes (Fig. 6), implicating decarboxylation-linked buffering in the response. Although such mechanisms are well documented in other bacteria, they have not been reported for *C. glutamicum*. Functionally, a *lysA* overexpression mutant exhibited enhanced survival under sustained low-pH stress, supporting a protective role, while *Cgl1110* and *fdh* remain candidate contributors pending experimental validation.

Marked low-pH regime specificity was observed, with only 252 DEGs forming the shared core between acute shock 1 h and sustained-stress 1 h responses (27.8% and 39.2%; Fig. 3), underscoring the distinct nature of short-term shock *versus* long-term adaptation. One key discrepancy lies in the regulation of *cglK* encoding a high-capacity K^+^ uptake channel that tunes Δѱ, supports pH homeostasis, and maintains osmotic balance. A previous study has shown that *ΔcglK* is highly sensitive to acidic conditions due to impaired K^+^ uptake and disrupted pH homeostasis [10]. In our dataset, *cglK* expression remained unchanged after acute shock but increased under sustained stress (Fig. 5B), likely reflecting post-translational control through energy and second-messenger signals (e.g., ATP/ADP, c-di-AMP). After acute shock, cells may stabilise Δψ by deploying pre-existing K^+^ transporters *via* post-translational control while energy charge is low. Under sustained low-pH stress, as ATP/ADP recovers, transcriptional upregulation of *cglK* expands K^+^ influx capacity, which in turn stabilises Δψ, offsets Na^+^ load from titration, and maintains turgor. However, because bacterial K^+^ homeostasis is tightly regulated and intracellular K^+^ strongly affects Δψ and ΔpH, osmotic balance, and bioenergetics, *cglK* overexpression may also be detrimental especially under sustained stress, by driving excessive K^+^ uptake that overcompensates Δψ and destabilising ionic balance and membrane energetics, thereby reducing acid tolerance (Fig. 5C). Therefore, *cglK* expression should be dynamically tuned to external pH, ensuring controlled K^+^ influx that stabilises Δψ without causing overcompensation or osmotic stress.

Differential regulation was also evident in Na^+^/H^+^ transport systems. Acute low-pH shock rapidly induced *nhaP* and Na^+^/H^+^–dicarboxylate symporters (*Cgl2551*, *Cgl3027*, *dctA*), while repressing *mrp* (Fig. 5). At 1 h of sustained stress, *Cgl2551*, *Cgl1258*, *Cgl0206*, and *Cgl3027* were repressed, *Cgl1005* and *nhaP* were induced, and *mrp* genes were also repressed. This pattern likely prevents excessive Na^+^ loading and reduces proton influx, thereby preserving PMF and avoiding osmotic stress during initial adaptation. As metabolism was re-established by 18 h of sustained stress, concurrent induction of *nhaP*, *mrp* operons, and additional symporters suggests a hierarchical transition from low-capacity exchangers to high-capacity Mrp systems, fine-tuning ΔpH and Δψ as pHᵢ and ATP/ADP recovered. These data delineate a temporal hierarchy of antiporter engagement not previously resolved in *C. glutamicum*.

Energetics and redox homeostasis diverged sharply between regimes. Acute low-pH shock rapidly repressed central metabolism and the *bc*_1_-*aa*_3_ aerobic respiration branch, reflecting an immediate collapse of energy charge; while under sustained low-pH stress, the PPP was activated to generate NADPH for antioxidant defence, lower glycolysis and *sucCD* provided substrate-level ATP, and *ppc* supported anaplerosis. Respiration shifted toward the cytochrome *bd* oxidase (*cydAB*) branch, which operates efficiently under proton-rich and microaerobic conditions owing to its high oxygen affinity and greater robustness than the *bc*_1_-*aa*_3_ supercomplex, while translocating fewer protons and producing less ROS. In parallel, induction of the nitrate-respiration genes (*narGHIJ*, *narK*) provided an auxiliary electron sink that reoxidised the menaquinone pool when aerobic oxidases were downregulated. This redox diversion prevents semiquinone accumulation and associated ROS formation while maintaining a modest PMF. Overexpression of *narIJK* further improved survival at low pH, likely by sustaining electron flow and redox balance, thereby avoiding quinone over-reduction and preserving ATP turnover (Fig. 4). Together, these adjustments define an energetically conservative, redox-stabilising respiratory strategy that underpins long-term adaptation of *C. glutamicum* to acidic environments.

## 5 Conclusions

By integrating time-resolved RNA-seq, viability assays, and genetic validation, this study provides the first systems-level dissection of how *Corynebacterium glutamicum* adapts to acute *versus* sustained low-pH stress. Both regimes activated a shared early defence network, yet sustained acidity triggered a distinct energetic and redox reprogramming centred on ion transport, nitrate respiration, and metabolic buffering. Newly uncovered protective modules including β-ketoadipate catabolism, urease-mediated ammonium release, Na^+^/H^+^ antiport, and nitrate respiration were functionally validated as contributors to acid tolerance. These findings extend current understanding of low-pH adaptation in *C. glutamicum*, revealing how proton extrusion, metabolic buffering, and redox-balancing pathways act in concert to stabilise intracellular pH and sustain energy homeostasis. The shock-*versus*-sustained framework developed here provides a useful basis for guiding future efforts to improve strain robustness and process stability under acidic fermentation conditions.

## CRediT authorship contribution statement

**Yuqing Hao:** Visualization, Methodology, Investigation. **Jianyu Xu:** Visualization, Writing – original draft, Writing – review & editing. **Bo Sun:** Investigation. **Hongyu Zhang:** Methodology. **Huifeng Gu:** Investigation. **Yongjian Wang:** Writing – review & editing. **Ping Zheng:** Methodology. **Kaizhi Jia:** Conceptualization. **Yan Zhu:** Validation, Supervision, Methodology, Investigation, Conceptualization, Writing – original draft, Writing – review & editing. **Jibin Sun:** Conceptualization.

## Declaration of competing interest

The authors declare that they have no known competing financial interests or personal relationships that could have appeared to influence the work reported in this paper.

## Figures and Tables

**figs1:**
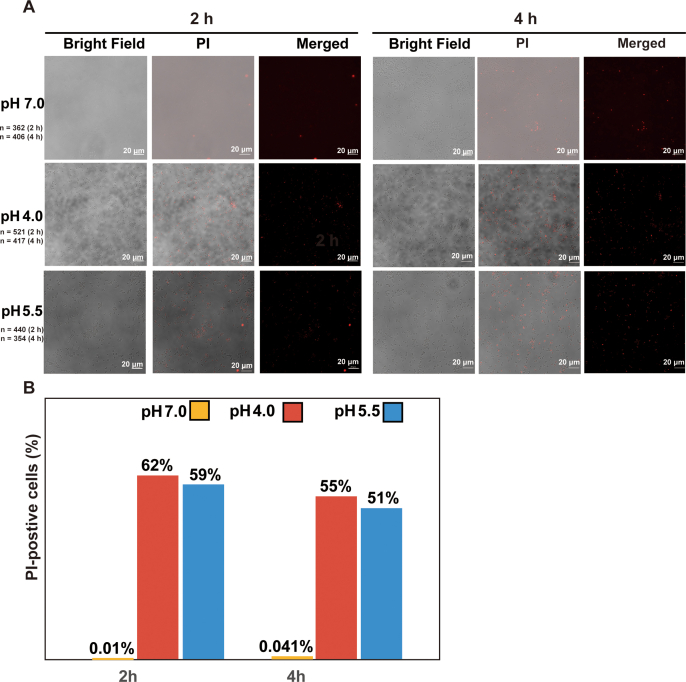


**figs2:**
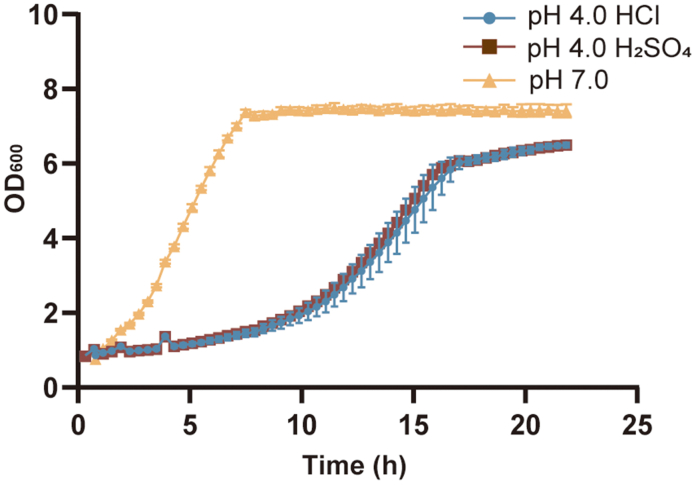


**figs3:**
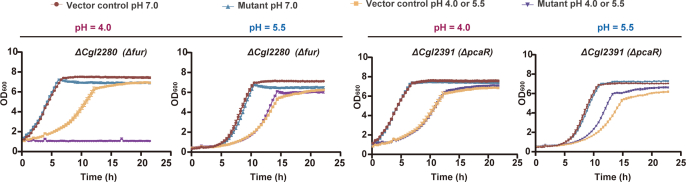


## Data Availability

The raw sequencing information have been deposited in Bioproject database under accession number PRJNA1358764, and the corresponding analysis scripts are available from the corresponding author upon reasonable request.
